# Successful Peripheral Blood Stem Cells Collection in Imatinib Pretreated and Nilotinib-Treated Chronic Myeloid Leukemia Patient

**DOI:** 10.1155/2010/460859

**Published:** 2010-03-04

**Authors:** Samuel Vokurka, Vladimir Koza, Daniel Lysak, Michal Karas, Pavel Dvorak, Pavel Jindra, Marcela Hrabetova, Vera Vozobulova

**Affiliations:** ^1^Department of Haemato-Oncology, University Hospital in Plzen, Plzen 304 60, Czech Republic; ^2^Department of Medical Genetics, University Hospital in Plzen, Plzen 304 60, Czech Republic

## Abstract

We report a case of a successful mobilization and harvest of the peripheral blood stem cells (PBSCs) in imatinib-pretreated and nilotinib treated 52-year-old woman diagnosed with Philadelphia chromosome-positive and BCR-ABL (b2a2) positive chronic phase CML in 2/2002. She failed interferon-alfa and imatinib treatment. She achieved her first complete molecular remission after 16 months of nilotinib treatment and later on was mobilized with filgrastim at a dose of 10 ug/kg/day applied subcutaneously once daily. The total number of 2.98 × 10^6^ CD34+ cells/kg was harvested on the fourth day of the mobilization. The autologous graft of the stem cells was cryopreserved and tested for the residual disease: the FISH revealed negative results and the RT-PCR was positive (BCR-ABL/ABL ratio 0,0017 in RQ-PCR). To our knowledge, this is the first report of successful PBSC harvest in a patient significantly pretreated with imatinib and nilotinib.

##  The Letter

The majority of patients diagnosed with chronic phase chronic myeloid leukemia (CML) can expect to have durable responses (88% OS at 6 years) with good quality of life on imatinib treatment [1]. Allogeneic stem cells transplantation (SCT) is no longer considered as the first line treatment in such patients and since 1999 there was a considerable reduction in the numbers of these transplants reported to the European Group for Blood and Marrow Transplantation—EBMT [2]. Autologous SCT might decrease the disease progression, offer valuable prolongation of survival for selected groups of patients, and can restore susceptibility to imatinib [3]. According to EBMT recommendations, however, this treatment strategy remains considered as developmental in chronic and accelerated CML phase [3, 4]. The number of autologous SCT for CML has been very low recently and only 6 cases were reported to the EBMT in 2007 [5]. On the other hand, clinical trials showed feasibility and efficacy of collection and storage of peripheral blood stem cells (PBSCs) from imatinib-treated patients in complete cytogenetic remission [6, 7]. However, experience with harvesting PBSC in nilotinib-treated CML patients is lacking. 

Nilotinib (AMN107, Tasigna-Novartis), a second generation tyrosine-kinase inhibitor and aminopyrimidine derivative of imatinib with activity against Arg, Kit, and platelet-derived growth factor receptor (PDGFR), but not Src-family kinases, is 10 to 50 times more potent than imatinib in inhibiting the proliferation and autophosphorylation of wild-type BCR-ABL cell lines and most of the BCR-ABL mutants, except the T315I mutant [8]. Predominant effect of nilotinib is antiproliferative rather than proapoptotic and does not induce apoptosis in primitive CD34+ CML cells; whereas those more mature are beginning to undergo apoptosis [9]. In chronic phase CML patients, resistant or intolerant to imatinib, nilotinib leads to 44% complete cytogenetic remissions (CCyR) that are durable with estimated 2 years OS 88% [10].

Here we report a case of successful mobilization and harvest of PBSC in imatinib pretreated and nilotinib treated 52-year-old woman with no related stem cells donor available and reluctant to undergo an alternative donor search. The patient was diagnosed with Philadelphia chromosome-positive and BCR-ABL (b2a2) positive chronic phase CML, presenting as intermediate prognostic group both sec. Sokal and Hasford, in 2/2002. Due to interferon-alfa resistance, imatinib 400 mg/day was started in 7/2002.The first CCyR was achieved twenty months later. Unfortunately, the cytogenetic relapse occurred in 3/2006. Due to further disease progression, despite the imatinib 600 mg/day escalation, nilotinib was started 400 mg twice daily within the CAMN107A Novartis Pharmaceuticals open-label ENACT trial in 12/2006. The second CyCR was achieved after 6 months (in 6/2007) and the first complete molecular remission (CMoR) after 16 months (in 4/2008) of the nilotinib treatment. In 2/2009, while being still in her first CMoR, the patient agreed on the autologous PBSC mobilization.

The patient was mobilized with filgrastim (Neupogen, Amgen, USA) at a dose of 10 ug/kg/day applied subcutaneously once daily for four days. The CML treatment was not interrupted and nilotinib was administered to the patient during the mobilization and harvest. The baseline and the apheresis day (the fourth day of the mobilization) values of the leucocytes, hemoglobin, and platelets were: 7,0 and 44,3 × 10^9^/L, 140 and 134 g/L, 296 and 320 × 10^9^/L, respectively. The CD34+ count in peripheral blood at the day of the apheresis reached 62,0 cells/*μ*L (the enumeration performed by the ISHAGE-based protocol on the Coulter XL flow cytometer). The PBSC apheresis was performed by means of continuous-flow blood cell separator (COBE Spectra, Caridian BCT, USA) via dialysis catheter inserted into femoral vein. Acid citrated dextrose (ACD-A) was used as the anticoagulant. The processed blood volume ranged between two and three times of the patient's total blood volume (15 litres). The apheresis product contained 2,98 × 10^6^ CD34+ cell/kg patient's weight was subjected to controlled rate freezing (IceCube, SY-LAB, Austria) and stored in liquid nitrogen under standard conditions. The product was also tested for the residual disease: the FISH revealed negative results, the RT-PCR was positive (BCR-ABL/ABL ratio 0,0017 in RQ-PCR). 

The mobilization and harvest complications included elevated body temperature up to 37,5 degrees centigrade, moderate musculoskeletal pains, headache and mild citrate toxicity with paresthesias. In 10/2009, the patient keeps on nilotinib treatment 400 mg twice daily (35 months duration) and remains in CMoR with good quality of life for almost eight years since the diagnose. 

As for the methodology of the laboratory testing, the BCR-ABL transcripts were measured in bone marrow samples using end-point nested real-time PCR (RT-PCR) with minimum sensitivity of 1 : 1000000 and real-time quantitative PCR (RQ-PCR) for BCR-ABL and ABL genes by means of FusionQuant^R^ Kit (Ipsogen, France) on Light Cycler 1.2 platform with minimum sensitivity of 1 : 100000. Complete molecular remission (CMoR) was defined as no detectable transcripts both in RT and RQ-PCR. The Fluorescence in situ hybridization (FISH) analysis was performed to detect BCR-ABL fusion gene using the Vysis LSI BCR/ABL ES Dual Color Translocation Probe Set (Abbott Molecular Inc., USA) with the sensitivity of 1 : 200.

To our knowledge, this is the first report of successful PBSC harvest in a patient significantly pretreated with imatinib and nilotinib. The procedure was feasible with only mild and expected side effects. Respecting the mode of nilotinib action and concentration of CD34+ cells within the autologous graft, presence of BCR-ABL positive cells is likely to be anticipated even in a patient in CMoR. In the Bhatia et al. [7] cohort of imatinib patients, only 8% of 134 autologous PBSC collections were PCR negative. Given the feasibility of PBSC collection in imatinib and nilotinib-treated patients, with the individual role of the autologous SCT in CML treatment, the PBSC collections should be considered in otherwise transplantable patients without available and suitable donor of allogeneic hematopoietic stem cells.
